# Responding to the Challenges of Aging: A Review and Outlook on China’s Dementia Care Policy Since 2011

**DOI:** 10.1093/geroni/igaf122.2233

**Published:** 2025-12-31

**Authors:** Jiakang Zhu, Qingwei Wang, Jiawei Cao

**Affiliations:** The University of North Carolina at Chapel Hill, Chapel Hill, North Carolina, United States; Xi’an Jiaotong-Liverpool University, Suzhou, Jiangsu, China (People’s Republic); Nanjing University of Chinese Medicine, Nanjing, Jiangsu, China (People’s Republic)

## Abstract

China is facing a pressing public health challenge as its population ages rapidly, leading to a significant rise in dementia prevalence. This article systematically reviews China’s dementia care policies to identify gaps and provide recommendations for future policy design. We examine three key years—2011, 2016, and 2024—as benchmarks for shifts in policy focus and implementation. In 2011, dementia was officially recognized in policy documents, marking the beginning of governmental attention towards this growing issue. Early policies aimed to improve detection rates and provide financial subsidies to families affected by dementia. However, these measures fell short of addressing comprehensive care. By 2016, the introduction of the Healthy China 2030 plan emphasized the need for effective dementia interventions, aligning with the World Health Organization’s Dementia Global Action Plan. Despite these advancements, the implementation of policies encountered practical challenges. By 2024, the Chinese government incorporated proactive dementia prevention and treatment interventions into its five-year plans. This effort culminated in the release of the most comprehensive action plan to date, representing an important milestone in China’s approach to manage dementia. Our analysis identifies two major issues: an unclear delineation of departmental responsibilities and inconsistency terminology used in policy documents. The evolution of China’s dementia care policies provides valuable lessons for other aging societies confronting similar challenges. Clearer roles and a more consistent language are crucial for effective policy implementation in the future.

